# Prognostic and clinicopathological significance of tumor-stroma ratio in head and neck squamous cell carcinoma: A systematic review

**DOI:** 10.4317/medoral.24922

**Published:** 2022-06-19

**Authors:** Everton Freitas de Morais, Hannah Gil de Farias Morais, Hélder Domiciano Dantas Martins, Leonardo Magalhães Carlan, Antônio de Lisboa Lopes Costa, Roseana de Almeida Freitas

**Affiliations:** 1 DDS, MSc, PhD Student, Postgraduate Program in Oral Science. Federal University of Rio Grande do Norte, Natal, Rio Grande do Norte, Brazil; 2 DDS, MSc Student, Postgraduate Program in Oral Science. Federal University of Rio Grande do Norte, Natal, Rio Grande do Norte, Brazil; 3 DDS, MSc, PhD Student, Postgraduate Program in Dentistry. Federal University of Paraiba, João Pessoa, Paraiba, Brazil; 4 PhD in Oral Pathology. Professor of the Postgraduate Program in Oral Science. Federal University of Rio Grande do Norte, Natal, Rio Grande do Norte, Brazil

## Abstract

**Background:**

Analysis of the tumor microenvironment has been proposed as a strategy for the treatment and prognosis of different neoplastic processes. A grading system based on the tumor-stroma ratio (TSR), which evaluates the proportion of stroma in relation to neoplastic parenchyma at the invasion front, has shown a strong prognostic value in different neoplastic processes. The aim of the present systematic review was to understand the role of the TSR in head and neck squamous cell carcinoma (HNSCC), evaluating its correlation with clinical and prognostic parameters.

**Material and Methods:**

An electronic search was performed in PubMed/Medline, Web of Science, Science Direct, Scopus, Embase, and the Cochrane Collaboration Library. Publications assessing the relationship between TSR and prognosis in cases of HNSCC were eligible. The quality of the studies was assessed independently by four evaluators using the Newcastle-Ottawa scale.

**Results:**

After application of the previously es+lished inclusion/exclusion criteria, nine articles were included in the qualitative synthesis. With regards to quality on the Newcastle-Ottawa scale, an overall value of 4.55 was obtained. This systematic review demonstrated a strong association between TSR and prognosis in esophageal and oral squamous cell carcinomas.

**Conclusions:**

Histopathological analysis of the TSR can optimize the analysis of the prognosis of cases diagnosed with HNSSC. In addition, the TSR is a reliable and simple parameter that can be evaluated in hematoxylin/eosin-stained slides during routine laboratory examinations, showing high inter- and intraobserver agreement.

** Key words:**Head and neck cancer, squamous cell carcinoma, grade prognosis, tumor-stroma ratio.

## Introduction

Head and neck cancers are the sixth most common type of cancer and comprise a range of malignant neoplasms that affect the oral cavity, pharynx, larynx, esophagus, paranasal sinuses, and nasal cavity (1,2). The most common histopathological type of head and neck cancer, diagnosed in about 90% of cases, is squamous cell carcinoma (SCC), a malignant neoplasm that arises from the lining epithelium and is characterized by an aggressive biological behavior and high rates of invasion and metastasis (1,3). Squamous cell carcinoma often has a strong negative impact on the quality of life of affected patients because of treatment sequelae and low responsiveness to treatment, in addition to high mortality rates in which the 5-year survival rate is generally less than 50% (3-5).

In recent decades, cancer research has focused mainly on the tumor parenchyma by elucidating the role of different biomarkers that are involved in carcinogenesis. Currently, there is an increasing interest in the stromal component of the tumor considering the biological and chemical phenomena that support, nourish and protect the tumor parenchyma, which may favor tumor progression and resistance to antineoplastic treatment (6-10). The tumor microenvironment is rich and diverse and different types of cells can be identified, including cancer-associated fibroblasts, endothelial cells, pericytes, and immune cells (5,11). Thus, analysis of the tumor microenvironment has been proposed as a strategy for the treatment and prognosis of different neoplastic processes (12).

To quantify the stromal component and its interaction with tumor nests, recent studies have proposed some parameters that are measurable in hematoxylin/eosin-stained histological material (6,9,13). Among these parameters, the proportion between neoplastic cells and the tumor-associated stroma in tumor tissue was defined as the tumor-stroma ratio (TSR). The TSR assesses the proportion of stroma in relation to the neoplastic parenchyma at the invasion front (represent the deepest point of invasion).

Morphologically, tumor invasion front reflects various molecular interactions that are crucial for the progression of cancer and the analysis of morphological features of the invasion front demonstrated prognostic value as a supplement to the TNM (3). A high proportion of stroma in the invasion front is associated with an unfavorable clinical outcome and has been found to be an adverse prognostic factor for various tumors, including colorectal, esophageal, breast, endometrial, ovarian epithelial, cervical and hepatocellular carcinoma, as well as head and neck carcinoma (6,12).

In an attempt to understand the role of the TSR in head and neck squamous cell carcinoma (HNSCC), the main objectives of the present systematic review were: 1) to identify the possible relationship between TSR and different prognosis-related clinicopathological features; 2) to understand the role of TSR as a possible histopathological predictor of recurrence rates in cases of HNSCC, and 3) to evaluate the role of TSR as a possible histopathological predictor of survival in cases of HNSCC.

## Material and Methods

This systematic review was conducted according to the guidelines of the Preferred Reporting Items for Systematic Reviews and Meta-Analyses (PRISMA) (14). The study was registered with PROSPERO under number CRD42020211807.

- Search strategy and study selection

Searches were performed in PubMed/Medline, Web of Science, Science Direct, Scopus, Embase and the Cochrane Collaboration Library ​​(last update in October 2021). For searching the grey literature, OpenGrey and Google Scholar were also assessed. The strategy adopted sought to rescue as many studies as possible related to the subject. Boolean operators AND, OR, NOT was used, as described in Table 1. All references obtained were exported to EndNote Web™ (Thomson Reuters™, Toronto, Canada) software, in which duplicated records were removed.

In addition, the reference lists of potentially eligible articles were hand searched. Duplicates were identified and removed. The search was performed without time or language restrictions. Manuscripts not originally published in English were translated for subsequent assessment.

**Figure 1:**
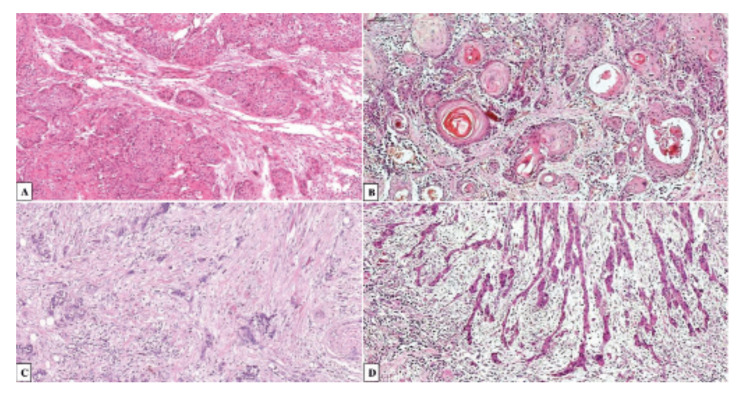
Hematoxylin and eosin stained 5 µm sections of oral squamous cell carcinoma. Tumor cells were present at all borders of the image field. (A-B) TSR ≤ 50%, stroma-poor. (C-D) Selection of the area with the highest proportion of stroma.

The present review focused on the following research question: What is the possible relationship between TSR and the clinicopathological characteristics and prognosis of HNSCC? Studies that met the following eligibility criteria according to the PICO framework were included in this systematic review: 1) Patients: patients diagnosed with HNSCC; 2) Intervention: HNSCC cases analyzed histologically and graded according to TSR; 3) Control: the groups analyzed were classified as high and low TSR; 4) Outcome: recurrence rate and disease-specific and overall survival of the HNSCC cases analyzed; 5) Design: the included studies had a retrospective cohort design. The publications were considered eligible when they met the following inclusion criteria: 1) the study evaluated the relationship between TSR and the prognosis of the sample of HNSCC cases by analyzing, for example, overall and/or disease-specific survival; 2) the patients with HNSCC were divided into two groups: low TSR (stroma-rich) in which the percentage of tumor stroma is ≥ 50% and high TSR (stroma-poor) in which the percentage of tumor stroma is < 50% (Fig. 1). Meanwhile, exclusion criteria were: 1) Review articles, conference abstracts, editorials and letters, 2) studies that did not investigate the TSR in HNSCC; and 3) deficient clinicopathological data (e.g., absence of data related to clinical tumor staging, lack of description on how the TSR evaluation process was performed).

**Table 1:**
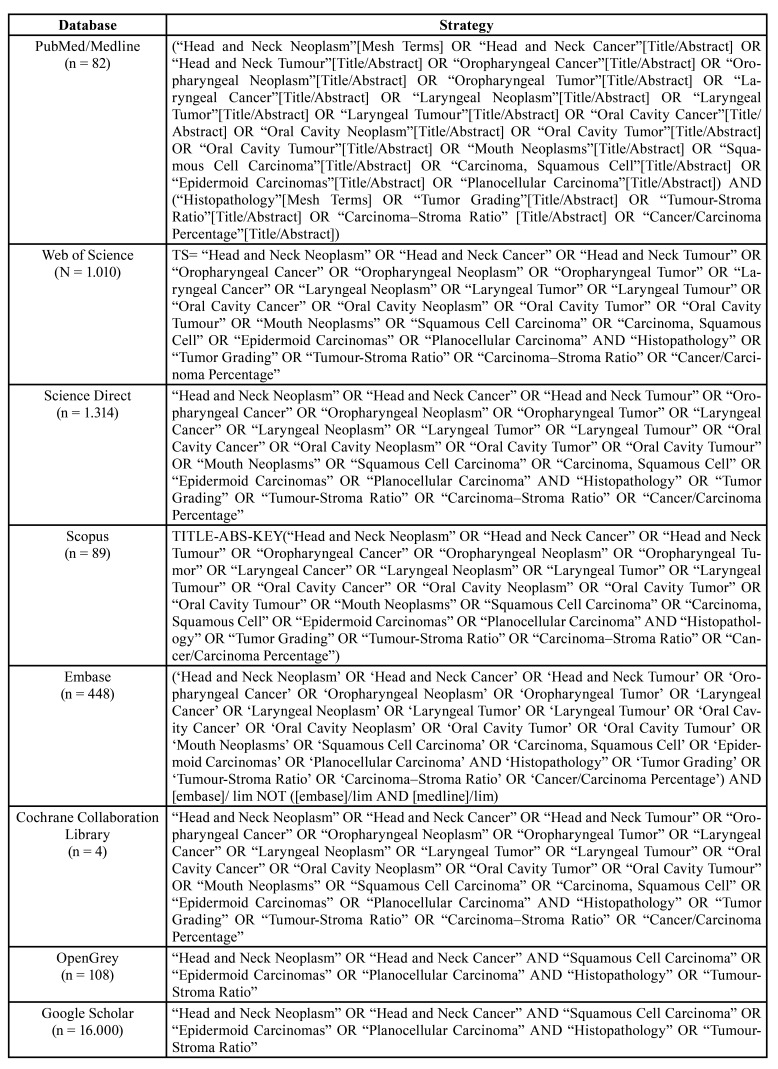
Search strategy performed at the databases until Oct 2021.

- Data extraction and Analysis

Four evaluators reviewed and independently extracted the data from all eligible studies based on the selection criteria. During the selection process, any disagreement between reviewers was resolved in a consensus meeting. The following data were extracted: name of the authors, year of publication, region of the study, follow-up time, sample size, clinicopathological characteristics (e.g., histopathological grade and clinical stage), TSR, treatment, recurrence and survival rates, and hazard ratio (HR).

Since only cohort studies were included, the quality of the study was assessed independently by four evaluators using the Newcastle-Ottawa scale (15). Disagreements were resolved in a consensus meeting.

## Results

- Study selection and characteristics

**Figure 2:**
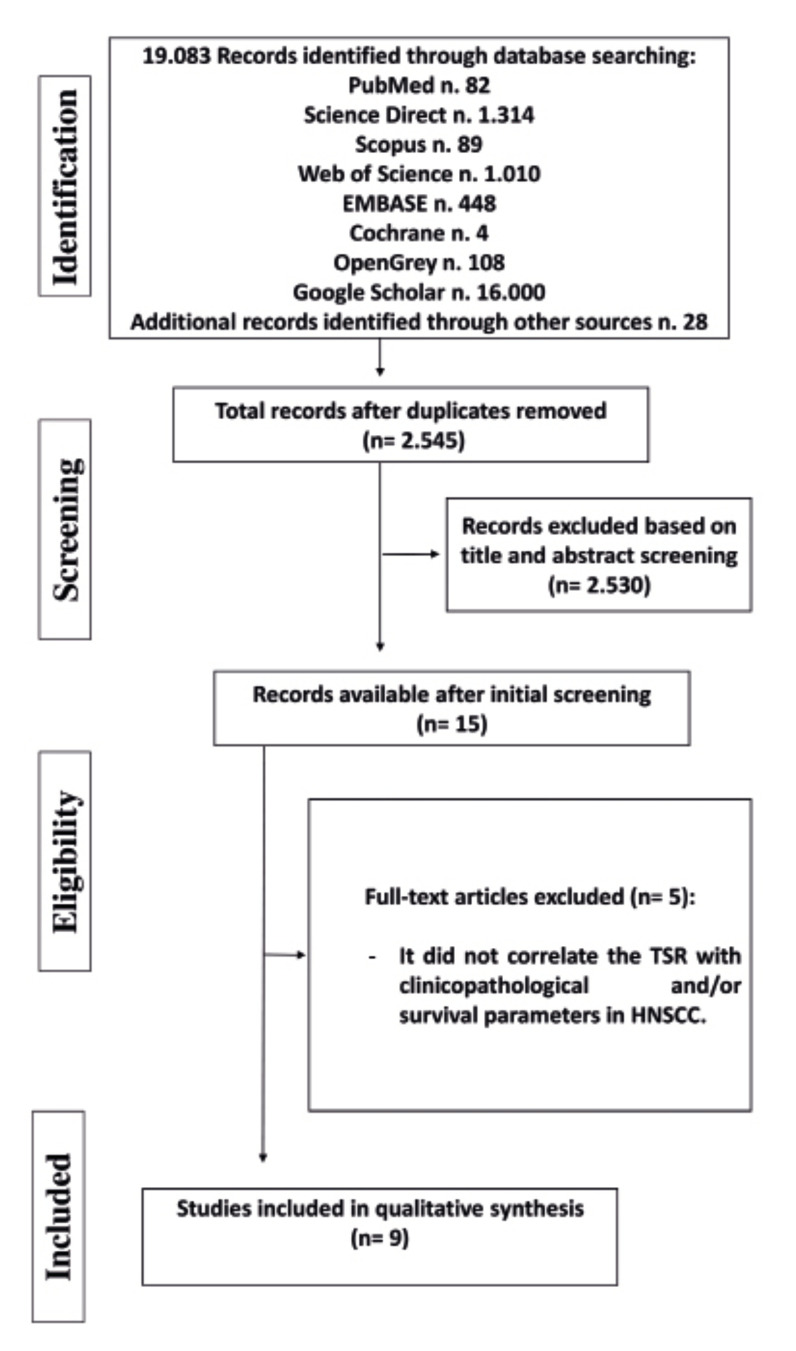
Flow diagram showing the electronic and manual search strategy for article selection.

The search strategy of this systematic review retrieved 19,083 studies from the different databases analyzed. After initial screening of the titles and abstracts, 15 studies were considered potentially eligible and the full text was read by four evaluators (EFM, HGFM, LMC, HDDM). After application of the previously established inclusion/exclusion criteria, nine articles were included in the present systematic review (8,9,13,16-20). Fig. 2 shows the flow chart illustrating the article screening and selection process.

The included studies analyzed samples of SCC affecting different anatomical regions of the head and neck: esophagus (16), larynx (13,17), oral cavity (8,9,19-21), and pharynx (13). Considering all selected studies, a total of 1,367 patients were analyzed (mean of 151.8 patients per study). Among the studies with available data, there was a slight predominance of male patients and most of the patient were in their fifth to seventh decade of life.

Regarding clinical stage, Almangush *et al*. (19) exclusively evaluated cases diagnosed in early stages (T1 and T2), while the proportion of stages III and IV was higher in the remaining studies. Surgery was the predominant treatment in all studies. Table 2 and Table 3 show the clinicopathological characteristics of the patients and of the tumor in the selected studies. The results of quality assessment of the selected studies are shown in Table 4.

The methods for the analysis of TSR were extracted from the selected studies. Briefly, the tumor invasion front was first identified by microscopic analysis at the lowest magnification. Next, the area of the invasion front with the highest proportion of tumor stroma was identified and a score was attributed to each case evaluated at a higher magnification. Eight of the selected studies dichotomized the groups into 1) a stroma-rich group defined as a high proportion of stroma and a low proportion of tumor cells (TSR ≥ 50%), and 2) a stroma-poor group corresponding to a low proportion of stroma and a high proportion of tumor cells (TSR < 50%) (8,9,13,16,17,19-21). Only Choi *et al*. (18) did not dichotomize the cases analyzed according to TSR. All included studies evaluated the proportion of stroma in hematoxylin/eosin-stained slides.

- Correlation between TSR and clinicopathological features in head and neck squamous cell carcinoma

The relationship between TSR and clinicopathological parameters was analyzed in different studies. Niranjan *et al*. (20) observed an association between stroma-rich tumors (low TSR) and invasion depth (> 10 mm) and invasion pattern (perineural and vascular) of the tumor (*p* < 0.05). Almangush *et al*. (19) studied only patients diagnosed in early stages; 89 cases (28.6%) were classified as stroma-rich and 222 (71.4%) as stroma-poor. There was no association between TSR and age sex, cTNM stage or histological grade according to the model proposed by the WHO (*p* > 0.05). However, the TSR was associated with perineural invasion (*p* = 0.04).

In the study of Dourado *et al*. (21), the TSR was significantly associated with smoking (*p* = 0.04) and location of the primary tumor (*p* = 0.002) in oral SCC cases. Also evaluating oral SCC cases, Rani *et al*. (9) observed a significant correlation between TSR and size of the primary tumor (*p* = 0.001). However, the study of Mascitti *et al*. (8) reported no association between clinicopathological parameters and TSR. Likewise, Ünlü *et al*. (17) found no association of TSR with the clinical parameters analyzed, including tumor location, histological grade, clinical stage, or perinodal invasion.

- Correlation between TSR and recurrence and survival in head and neck squamous cell carcinoma

The selected studies reported conflicting results regarding the correlation between TSR and patient survival, in which stroma-rich tumors were associated with poor survival.

**Table 2:**
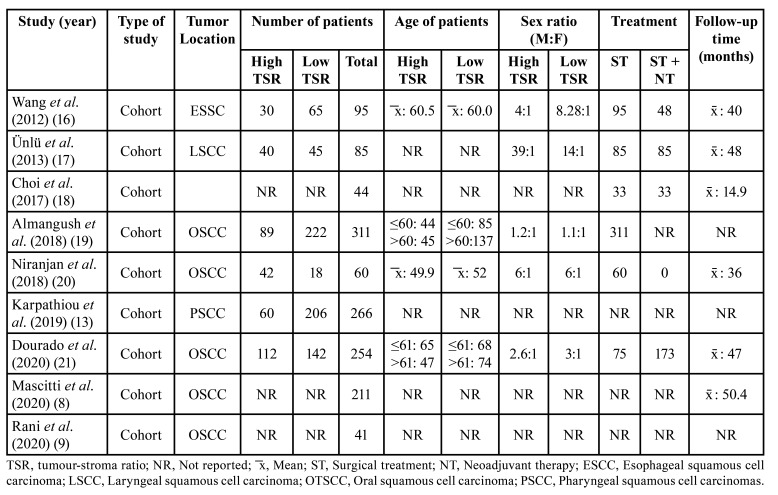
Patient characteristics in studies included in the qualitative synthesis stratifed by tumour-stroma ratio.

**Table 3:**
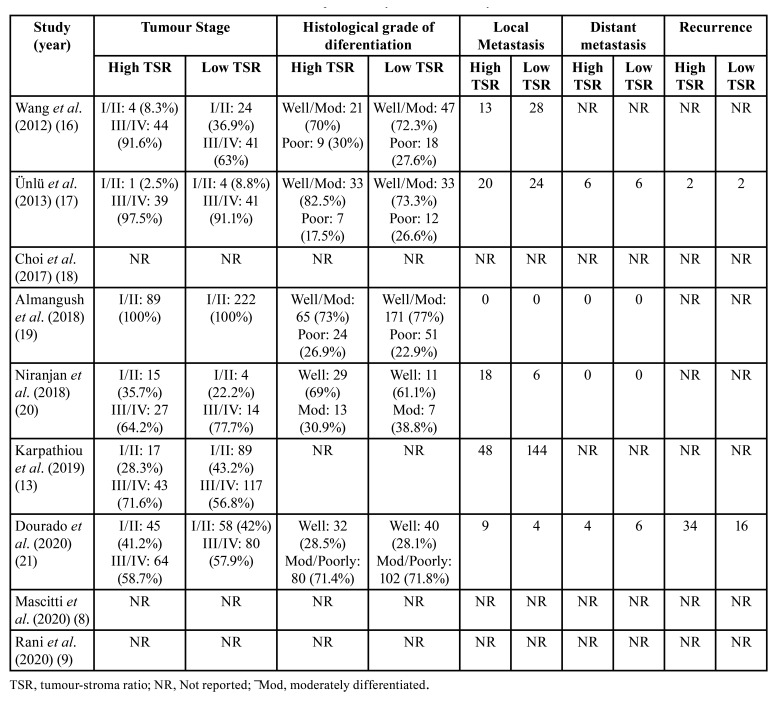
Tumour characteristics in studies included in the qualitative synthesis stratifed by tumour-stroma ratio.

**Table 4:**
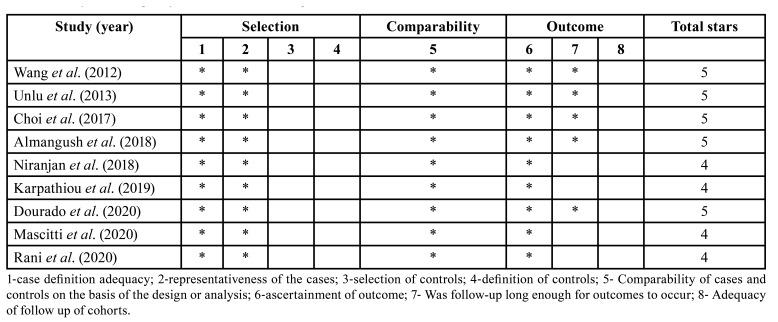
Analysis of the quality of the studies according to the Newcastle-Ottawa scale.

In the study of Wang *et al*. (16), the median overall survival of patients in the stroma-poor group was 39 months (95% CI, 36-43 months) compared to 24 months (95% CI, 18-29 months) in the stroma-rich group. The TSR was also an independent prognostic factor for 3-year disease-free survival. Ünlü *et al*. (17) conducted the first study that analyzed the TSR in laryngeal SCC cases; however, analysis of overall or disease-free survival did not reveal a significant association with TSR (*p* = 0.08 and *p* = 0.38, respectively).

In a sample of oral SCC, Almangush *et al*. (19) observed that stroma-rich cases were associated with higher recurrence rates (*p* = 0.02) and poor overall and disease-free survival. In the study of Niranjan *et al*. (20) that also evaluated oral SCC cases, the median overall and disease-free survival was 40 months (95% CI, 38-41 months) in stroma-poor tumors compared to 38 months (95% CI, 36-39 months) in stroma-rich tumors.

Patients with tumors classified as stroma-rich developed significantly more local and regional recurrences than patients with stroma-poor tumors in the study of Dourado *et al*. (21). In that study, Cox multivariate analysis confirmed that the TSR (HR 2.56; 95% CI, 1.18-5.55; *p* = 0.017) was an independent prognostic marker of disease-free survival. These results agree with the findings of Mascitti *et al*. (8) who, using a Cox proportional hazards model, demonstrated that patients with stroma-rich oral SCC had a significantly lower disease-specific and overall survival than stroma-poor cases (*p* = 0.036 and *p* = 0.051, respectively).

## Discussion

The characteristics of the tumor stroma have received greater attention in recent studies on the tumor microenvironment (22,23). Within this context, there is evidence indicating an important role of the stroma in cancer development and progression by promoting tumor angiogenesis, the secretion of different growth factors, and resistance to antineoplastic treatment, thus favoring tumor invasion, metastasis, and recurrence (5,11,20).

The TSR can be easily quantified on conventional hematoxylin/eosin-stained paraffin sections at the invasive front of the tumor. TSR scoring is a reliable system that has the potential to be used in everyday practice. The procedure is highly replicable, with little intra-observer variation (24). As a limitation of the technique, TSR is better assessed in deeper biopsies where the invasive front including cancer cells growth pattern may be more clearly identified (25).

Within this context, Mesker *et al*. (26) proposed the analysis of TSR as an independent prognostic factor in colorectal carcinoma. Recently, several other studies have suggested the TSR to be a useful tool in the prognosis of different cancers, including breast (27,28), endometrial (29), ovarian epithelial (30), cervical (31), hepatocellular (32), and head and neck cancers (6,9,11,13,16,17,19,21).

Therefore, the present systematic review analyzed the potential of TSR as a prognostic predictor in HNSCC. Divergent results regarding the association between TSR and survival according to the anatomic location of SCC have been reported. For example, in esophageal and oral SCC, stroma-rich tumors were associated with significantly lower survival when compared to stroma-poor tumors. The TSR was considered an independent prognostic factor for disease-free survival in esophageal SCC and was associated with high rates of locoregional recurrence in oral SCC (8,16,18,19-21). In contrast, no significant association between TSR and survival was observed in patients with laryngeal SCC (17).

This review also analyzed the association of TSR with clinicopathological parameters and found that a high proportion of stroma in oral SCC was significantly associated with the invasion depth and invasion pattern of the tumor (20), perineural invasion (19,20), location (21), and size of the primary tumor (9), indicating a poor prognosis. The reason for the poor outcome in patients with tumors that contain a higher stroma percentage is still unclear but is probably related to the interactions between tumor cells and the tumor microenvironment (21). The tumor-related stromal components are complex and include the extracellular matrix, various types of cells, and different secreted factors. According to Brooks *et al*. (33), the tumor stroma may act as a “barrier” to antitumor immune responses.

Cancer-associated fibroblasts are known to be related to proliferation and tumor progression since they synthesize and secrete pro-tumor growth factors, extracellular matrix proteins, cytokines, angiogenic molecules, and proteolytic enzymes (9,19,21). In addition, tumor-associated macrophages have also been shown to promote tumor growth and to inhibit antitumor immune responses (19). A more fibrotic tumor stroma may also inhibit the release of drugs to the tumor mass, facilitating chemoresistance. Thus, these combined features of the tumor stroma may explain why a stroma-rich tumor is more likely to exhibit an aggressive biological behavior, with a consequent negative outcome for the patient (21).

According to Almangush *et al*. (19) and Dourado *et al*. (21), the combination of TSR and tumor budding provided a hazard model with discrimination ability to predict the prognosis of patients with oral tongue SCC. This fact is probably due to the combination of independent prognostic parameters that significantly increase the prognostic power (21).

Regarding the evaluation of the risk of bias according to Newcastle-Ottawa Scale (15), all studies were identified with scores between 4-5 (medium quality). Some limitations of the present study and of those selected should be mentioned. First, since SCC is a malignancy with a heterogenous biological behavior, studies involving large samples according to the different anatomical locations are necessary to determine the definitive value of TSR for the prognosis of different carcinomas. The follow-up period of the selected studies was inconsistent, posing an imminent risk of bias. In addition, clinical treatment is also a significant prognostic factor in patients with cancer. It remains unknown whether the effect of TSR is independent of clinical treatment since the selected studied failed to analyze this parameter.

The TSR is a reliable and simple parameter that can be evaluated in hematoxylin/eosin-stained slides during routine laboratory examinations, showing high inter- and intraobserver agreement (8,11,19-21). However, additional studies are recommended to explore the clinical importance of the stroma related to tumor formation and development and possible therapeutic approaches that could use TSR as a target.

The present systematic review demonstrated a strong association between TSR and prognosis in esophageal and oral SCC. In addition, the TSR can be easily obtained during routine histopathological examination and its combination with parameters of cancer invasion such as tumor budding can provide a better prognostic value than individual assessment of each parameter. Within this context, future studies should explore the underlying mechanisms and interactions of the tumor stroma and its prognostic capacity since cellular and molecular elements of the tumor microenvironment are emerging as important therapeutic targets.
